# Perceptions and Attitudes Toward COVID-19-Related National Response Measures of Vietnamese: Implications for Pandemic Prevention and Control

**DOI:** 10.3389/fpubh.2020.589053

**Published:** 2020-10-09

**Authors:** Thao Thi Phuong Nguyen, Long Hoang Nguyen, Huong Thi Le, Giang Thu Vu, Men Thi Hoang, Diep Ngoc Nguyen, Xuan Thi Thanh Le, Bach Xuan Tran, Thao Thanh Nguyen, Quan Thi Pham, Nhung Thi Kim Ta, Quynh Thi Nguyen, Carl A. Latkin, Roger C. M. Ho, Cyrus S. H. Ho

**Affiliations:** ^1^Institute for Preventive Medicine and Public Health, Hanoi Medical University, Hanoi, Vietnam; ^2^Center of Excellence in Evidence-Based Medicine, Nguyen Tat Thanh University, Ho Chi Minh City, Vietnam; ^3^Institute for Global Health Innovations, Duy Tan University, Da Nang, Vietnam; ^4^Faculty of Pharmacy, Duy Tan University, Da Nang, Vietnam; ^5^Bloomberg School of Public Health, Johns Hopkins University, Baltimore, MD, United States; ^6^Department of Psychological Medicine, Yong Loo Lin School of Medicine, National University of Singapore, Singapore, Singapore; ^7^Institute for Health Innovation and Technology (iHealthtech), National University of Singapore, Singapore, Singapore; ^8^Department of Psychological Medicine, National University Hospital, Singapore, Singapore

**Keywords:** COVID-19, attitudes, perceptions, lockdown, Vietnam

## Abstract

**Introduction:** Public perceptions and attitudes toward preventive and control measures are vital to ensure the success of national response strategies in combating COVID-19. This study assessed perceptions and attitudes via the importance of national response measures to COVID-19 among people under the nationwide partial lockdown of Vietnam.

**Methods:** An online cross-sectional survey was conducted on 1382 people in Vietnam mainly public administration and health workers with relatives. Perceptions and attitudes toward seven national response measures to COVID-19 epidemics were assessed. Multivariable Tobit regression models were employed to identify factors associated with the perceptions.

**Results:** The proportion of participants strongly agreeing with the measure “Isolate people from abroad and people in contact with people infected with COVID 19” was the highest (96.9%), following by the measure “Obligatory to wear face masks in public places” (96.8%), and “Blockade of places having new cases” (92.9%). Living in the Southern region, having a family with more than 5 people, and having post-graduate education were negatively correlated to the levels of perceived importance of “Social distancing and community screening” measures. Meanwhile, having post-graduate education (Coef. = −0.04; 95%CI: −0.07; −0.01), working as white-collar workers (Coef. = −0.04; 95%CI: −0.08; −0.01), and having fixed-term, full-time employment (Coef. = −0.07; 95%CI: −0.10; −0.03) were inversely associated with the levels of perceived importance of the “Mandatory quarantine and personal protective equipment” measures.

**Conclusion:** This study informed highly positive perceptions and attitudes toward the national response measure to combat the COVID-19 in Vietnam. Contextualized strategies to maintain and improve these perceptions are warranted to ensure the success of preventive measures in the future.

## Introduction

COVID-19 pandemic has demonstrated its social, health, and economic devastations worldwide (1, 2). Until 19 July 2020, more than 14 million people in 216 countries and territories acquired severe acute respiratory syndrome coronavirus 2 (SARS-CoV-2), a cause of COVID-19, and approximately 600,000 COVID-19-related deaths are recorded (3). National lockdowns and other preventive measures such as social distancing, strict quarantine, contact tracing, or personal protective gear (e.g., face masks) promotion have been implemented across countries to control the source of infection, cut off the transmission route and mitigate the damage of the pandemic (4–6). Some initial progress has been achieved (3); however, many countries are still facing the risk of a recurring epidemic (7). Therefore, unless vaccines for this disease are available, these preventive measures continuously play an essential role in preventing the transmission in all nations (8).

Previous experiences indicated that the success of national response strategies heavily relied on the public's perceptions and attitudes toward the risk of an epidemic and the importance of preventive measures (9, 10). They have a major role in forming individuals' self-protection behaviors (11, 12), willingness to cooperate, and adopt new preventive measures during outbreaks (11–18). Lessons learned from the Severe acute respiratory syndrome (SARS) pandemic in 2003 showed the association between panic emotion and attitudes toward infectious diseases and preventive measures, which negatively affected the efforts to prevent the infectious transmission (9, 10). Prior literature also highlighted that the community's perceptions and attitudes were strongly related to the compliance toward preventive measures (19). Thus, assessing perceptions and attitudes toward national COVID-19 preventive measures are vital to understanding the sustainability of these strategies in the coming epidemics.

As being considered among countries with the highest risk of the COVID-19 pandemic, Vietnam has also implemented such preventive measures to combat the pandemic from very early phase (20). After detecting the first 16 positive cases, the Vietnam Government decided to tighten borders with China, revoked aviation licenses and restricted visas, warned citizens to avoid epidemic areas (21, 22), closure of all educational facilities (23), and call self-protective implementation (e.g., using facemasks, washing hands, and seeking medical care if getting symptoms) (24). From 7 March 2020, the Vietnamese Government decided 14-day compulsory quarantines for Vietnamese people returning from abroad, temporarily suspended visas for foreigners coming to Vietnam (25), and requested that people in the community report Covid-19 related health conditions (26). Moreover, closing all public areas except for essential facilities as well as bans and restrictions on public transport were implemented (27). On 1 April 2020, the 15-day national lockdown was performed as a strong response to prevent disease and reduce the transmission (28). With these strict measures, as a result, to date, only 332 positive cases were reported without any deaths (29), which has been much lower than other countries with similar population size (3).

Given the matter that Vietnam is still at risk of recurring COVID-19 pandemic (7), understanding the perceptions and attitudes of the community toward the national response measures for the COVID-19 is critically important for maintaining the success of Vietnam in controlling the epidemic. Thus, this study aimed to assess perceptions and attitudes toward the importance or necessity of national response measures to COVID-19 epidemics among people under the nationwide partial lockdown of Vietnam.

## Methods

### Study Setting and Participants

Data from an online cross-sectional survey were obtained within 1 week of the national lockdown in April 2020 due to the COVID-19 epidemic in Vietnam. During this period, all Vietnamese citizens were highly recommended to stay at home to prevent and control the COVID-19 outbreak. The respondents met the following inclusion criteria: (1) Aged 18 years or above; (2) Having ability to access the questionnaire on the web-based survey, and (3) Having ability to read and respond the questionnaire; and (4) Agreeing to take part in the research by approving the online informed consent.

The online survey was developed on the Survey Monkey platform, which had good usability, comprehensive feature sets, and qualified data storage and private information security. Links for the online survey were sent to respondents via email or social media platforms such as Zalo or Facebook. A snowball sampling method was applied in following steps: (1) First, we randomly recruited a core group including 80 staffs and 150 medical students at the Hanoi Medical University with different age and gender; (2) After completing the survey, they were asked to invited other Vietnamese people such as their family members, relatives, or friends in different provinces to participate in the survey. The study protocol was approved by the Institutional Review Board (IRB) of the Institute for Preventive Medicine and Public Health, Hanoi Medical University (Code 75/QD-YHDP&YTCC). A total of 1,382 respondents were enrolled in the study and they were mainly public administration and health workers with relatives.

### Measurement

The online questionnaire was developed by the research team to measure sociodemographic characteristics and perceptions about the importance of national response measures to the COVID-19 epidemic in Vietnam. The questionnaire was piloted with 10 staff and medical students at the Hanoi Medical University to ensure the logical order of items, language, and readability of the text in each question. The questionnaire was then revised according to the feedback of the participants and approved by the Institute for Preventive Medicine and Public Health, Hanoi Medical University. Collected information included:

- Socioeconomic status consisted of gender, region, age, family size, marital status, occupation, occupation status, and education level.
- Perceptions and attitudes about the importance of national response measures: Seven questions regarding perceptions about the importance of seven national response measures to COVID-19 epidemics were asked during the nationwide partial lockdown of Vietnam. Participants were asked to answer the questions: “To what extent do you agree or disagree with the importance and necessity of following measures to prevent and control COVID-19 epidemic in Vietnam?” with following seven items: (1) isolate people from abroad and people in contact with those infected with COVID-19; (2) obligatory to wear face masks in public places; (3) blockade of places having new cases; (4) implement social distancing; (5) close unnecessary services; (6) measure body temperature in public places; (7) close educational facilities.

Respondents evaluated the importance or necessity of these national response measures by using a five-level Likert scale (1 = strongly disagree, 2 = disagree, 3 = neutral, 4 = agree, and 5 = strongly agree).

### Data Analysis

STATA 15.0 software was used to analyze the data. Since education was a significant predictor for the attitude and perception toward COVID-19 epidemic and measures (30), descriptive statistics were used to examine the differences in the sociodemographic and perceptions among three education levels including “High school and below,” “Undergraduate,” and “Post-graduate.”

In this study, we applied an exploratory factor analysis (EFA) to evaluate the construct validity of the scales. We used the screen test to examine the threshold of eigenvalue, and a value of 1.0 was detected as a threshold for flattening out the curve. We also applied the Orthogonal Varimax rotation and Kaisers' normalization to reallocate seven items into two identified domains. A value of 0.4 was utilized as a cut-off threshold for factor loadings. The internal consistency reliability of each domain was evaluated by computing Cronbach's alpha. The score of each domain was computed by averaging the total score of all items in the domain. Domain's score ranges from 1 to 5. A higher score indicated higher level agreement about the importance/necessity of national response measures.

Given that data of domain scores were censored data, multivariable Tobit regression models were employed to identify factors associated with the perceptions about the importance/necessity of national response measures to COVID-19 epidemics. Independent variables included socioeconomic status (gender, region, age, family size, marital status, occupation, occupation status, and education level), while dependent variables were scores of two new domains, namely “Social distancing and community screening” and “Mandatory quarantine and personal protective equipment.” Stepwise forward selection strategies were utilized to produce the reduced models, with a *p*-value of log-likelihood test of 0.2 as a threshold for including variables into the model. *P* < 0.05 was defined as statistical significance.

### Ethical Consideration

The research was ethically approved by the Review Committee at Hanoi Medical University on March 27, 2020. The purpose of research and informed consent was provided online for participants to decide whether to participate. Participation was voluntary, and anonymity was assured. Respondents were able to decline to participate or withdraw from the online survey at any time.

## Results

### Socioeconomic Characteristics of Respondents

Table 1 shows that among 1,382 participants, 62.0% was female, 47.1% lived in Northern, and 76.7% married. The mean age was 36.4 (*SD* = 9.7) years old. There were 73.2% of respondents having from 3 to 5 people in the family. The majority of participants were health workers (69.7%), following by white-collar workers (10.4%), and students (6.7%). Most of the respondents had were public employees (64.7%).

**Table 1:** Socio-economic characteristics of respondents.

	**Education level**							
	**High school and below**		**Undergraduate**		**Post-graduate**		**Total**	
	***n***	**%**	***n***	**%**	***n***	**%**	***n***	**%**
**Total**	100	7.2	1,104	79.9	178	12.9	1382	100.0
**Gender**								
Male	35	35.0	399	36.1	91	51.1	525	38.0
Female	65	65.0	705	63.9	87	48.9	857	62.0
**Region**								
Northern	62	62.0	479	43.4	110	61.8	651	47.1
Central	31	31.0	434	39.3	37	20.8	502	36.3
South	7	7.0	186	16.8	29	16.3	222	16.1
Foreign people	0	0.0	5	0.5	2	1.1	7	0.5
**Age group**								
Under 25	65	65.0	48	4.3	0	0.0	113	8.2
25–34	10	10.0	506	45.8	40	22.5	556	40.2
35–44	11	11.0	342	31.0	67	37.6	420	30.4
Above 44	14	14.0	208	18.8	71	39.9	293	21.2
**Family size**								
1–2 people	24	24.0	170	15.4	20	11.2	214	15.5
3–5 people	68	68.0	807	73.1	137	77.0	1012	73.2
Above 5 people	8	8.0	127	11.5	21	11.8	156	11.3
**Marital status**								
Single	74	74.0	229	20.7	19	10.7	322	23.3
Married	26	26.0	875	79.3	159	89.3	1060	76.7
**Occupation**								
Health workers	15	15.0	854	77.4	94	52.8	963	69.7
Professional educators	1	1.0	37	3.4	31	17.4	69	5.0
White collar workers	1	1.0	114	10.3	29	16.3	144	10.4
Students	66	66.0	25	2.3	1	0.6	92	6.7
Others	17	17.0	74	6.7	23	12.9	114	8.2
**Type of contract**								
Public employment contract^*^	11	11.0	769	69.7	114	64.0	894	64.7
Permanent employment contract	9	9.0	152	13.8	28	15.7	189	13.7
Fixed-term fulltime contract^**^	6	6.0	107	9.7	16	9.0	129	9.3
No contract (Farmers/Students/Homemakers/ Unemployed/Retired)	70	70.0	46	4.2	6	3.4	122	8.8
Others	4	4.0	30	2.7	14	7.9	48	3.5
	**Mean**	***SD***	**Mean**	***SD***	**Mean**	***SD***	**Mean**	***SD***
**Number of children**	0.7	1.1	1.3	0.9	1.7	0.8	1.3	0.9
**Number of children aged above 15**	0.3	0.7	0.3	0.7	0.6	0.8	0.4	0.7
**Age**	28.0	11.9	36.2	9.0	42.1	8.7	36.4	9.7

* *Public employment contract: type of contract for people working in public non-business units under working contracts and salaried from salary funds of public non-business units in accordance with law*.

** *Fixed-term fulltime contract is a contractual relationship between an employee and an employer that lasts for a specified period. In Vietnam, there are two kinds of fixed-term contracts include the definite term contract of 12–36 months and the seasonal or fixed-term contract of <12 months*.

### Factor Loading of Exploratory Factor Analysis

Table 2 presents the EFA results. The proportion of participants strongly agreeing with the measure “Isolate people from abroad and people in contact with people infected with COVID 19” was the highest (96.9%), following by the measure “Obligatory to wear face masks in public places” (96.8%), and “Blockade of places having new cases” (92.9%). Two domains were reconstructed after the analysis including “Social distancing and community screening” (with 4 items, Cronbach's alpha = 0.74, mean = 4.7, *SD* = 0.5) and “Mandatory quarantine and personal protective equipment” (with 3 items, Cronbach's alpha = 0.68, mean = 5.0, *SD* = 0.2).

**Table 2:** Factor loading of exploratory factor analysis.

**Items**	**Strongly agree**		**Social distancing and community screening**	**Mandatory quarantine and personal protective equipment**
	***n***	**%**		
Isolate people from abroad and people in contact with people infected with COVID 19	1,339	96.9		0.85
Obligatory to wear face masks in public places	1,338	96.8		0.81
Blockade of places having new cases	1,284	92.9		0.69
Implement social distancing	1,068	77.3	0.80	
Close unnecessary services	1,063	76.9	0.80	
Measure body temperature in public places	1,015	73.4	0.57	
Close educational facilities	940	68.0	0.75	
**Cronbach's alpha**			0.74	0.68
**Mean (1–5)**			4.7	5.0
**SD**			0.5	0.2

### Perception of National Response Measures to COVID-19 Epidemics Among Respondents

Table 3 depicts that the score of each measure, expect “Close educational facilities” and “Isolate people from abroad and people in contact with people infected with COVID 19,” as well as the score of each domain were significantly different among three groups of education level (*p* < 0.05).

**Table 3:** Perception of national response measures to COVID-19 epidemics among respondents.

	**Education level**								
	**High school and below**		**Undergraduate**		**Post-graduate**		**Total**		***p*-value**
	**Mean**	***SD***	**Mean**	***SD***	**Mean**	**SD**	**Mean**	***SD***	
**Social distancing and community screening**	4.7	0.4	4.7	0.4	4.5	0.6	4.7	0.5	<0.01
Close educational facilities	4.6	0.5	4.6	0.7	4.6	0.7	4.6	0.7	0.60
Measure body temperature in public places	4.7	0.5	4.7	0.6	4.4	0.8	4.7	0.6	<0.01
Implement social distancing	4.7	0.6	4.7	0.6	4.6	0.7	4.7	0.6	0.02
Close unnecessary services	4.6	0.6	4.8	0.5	4.6	0.7	4.7	0.6	<0.01
**Mandatory quarantine and personal protective equipment**	4.9	0.2	5.0	0.2	4.9	0.2	5.0	0.2	<0.01
Obligatory to wear face masks in public place	5.0	0.2	5.0	0.2	4.9	0.3	5.0	0.2	0.01
Isolate people from abroad and people in contact with people infected with COVID 19	4.9	0.3	5.0	0.2	5.0	0.2	5.0	0.2	0.49
Blockade of places having new cases	4.9	0.3	4.9	0.3	4.8	0.4	4.9	0.3	<0.01

### Associated Factors With the Levels of Perceived Importance of National Response Measures to COVID-19 Epidemics

Table 4 reveals the factors associated with the perceived importance of national response measures to COVID-19 epidemics. People who were female (Coef. = 0.09; 95%CI: 0.04; 0.14), aged 35–44 years old (Coef. = 0.09; 95%CI: 0.03; 0.14) and married (Coef. = 0.07; 95%CI = 0.01; 0.13) had a significantly higher score in “Social distancing and community screening” domain compared with those who were male, aged under 25 years and single, respectively. Meanwhile, living in the Southern region, having a family with more than 5 people, and having post-graduate education were negatively correlated to the levels of perceived importance of this measure group.

**Table 4:** Associated factors with the levels of perceived importance of national response measures to COVID-19 epidemics.

**Characteristics**	**Social distancing and community screening**		**Mandatory quarantine and personal protective equipment**	
	**Coef**.	**95% CI**	**Coef**.	**95% CI**
Region (vs. Northern)				
Southern	−0.13^***^	−0.19; −0.06		
Foreign			−0.22^***^	−0.35; −0.08
Gender (vs. Male)				
Female	0.09^***^	0.04; 0.14	0.02^*^	−0.00; 0.04
Age group (vs. Under 25)				
35–44	0.09^***^	0.03; 0.14		
Number of children			−0.02^**^	−0.03; −0.00
Marital status (vs. Single)				
Married	0.07^**^	0.01; 0.13	0.03	−0.01; 0.06
Family size (vs. 1–2 people)				
Above 5 people	−0.10^***^	−0.18; −0.03		
Education level (vs. High school and below)				
Post-graduate	−0.18^***^	−0.25; −0.10	−0.04^***^	−0.07; −0.01
Occupation (vs. Health workers)				
White-collar workers			−0.04^***^	−0.08; −0.01
Others	0.07	−0.02; 0.15		
Occupation status (vs. Public employment contract)				
Fixed-term fulltime contract			−0.07^***^	−0.10; −0.03
No contract			−0.03	−0.07; 0.01

*** *p < 0.01*,

** p < 0.05, and

* *p < 0.1*.

In terms of the “Mandatory quarantine and personal protective equipment” measure group, people having post-graduate education level (Coef. = −0.04; 95%CI: −0.07; −0.01), living outside Vietnam (Coef. = −0.22; 95%CI: −0.35; −0.08), working as white-collar workers (Coef. = −0.04; 95%CI: −0.08; −0.01), and having full-time employment contracts (Coef. = −0.07; 95%CI: −0.10; −0.03) were inversely associated with the levels of perceived importance of the measures.

## Discussion

This study was performed during the national lockdown in Vietnam, which might be helpful to reflect the perceptions and attitudes of the general population toward national response measures to address the COVID-19 epidemic. Findings from this study showed a high level of agreement among the general population toward the importance and necessity of national response measures to combat the COVID-19 epidemic. Moreover, the result indicated regional variations in the perceived importance, as well as the drivers of these perceptions, such as gender, educational level, marital status, family size, occupation, and participants' employment status. The finding from our study can inform governments in implementing appropriate measures in the coming epidemics.

As being aware of challenges ahead regarding limited resources and healthcare capacities (31), the Government of Vietnam had implemented various mandatory measures in the early phase of the epidemic to “flattening the curve” (32). It is necessary for the government to have time for preparing a possible upsurge in the number of COVID-19 cases and forecasting the pandemic peak in the next stages (13, 33, 34). Findings from our study indicated a significantly high agreement about the importance of response measures that the Government performed during the epidemic. This phenomenon can be explained that along with the role of the Government's leadership, the transparency in epidemic information and the great involvement of mass media were critically important in developing the beliefs of the community in the Government's actions, and the importance of changing behaviors such as wearing masks, good hygiene, social distancing, or compulsory quarantine (32, 35, 36). Our study implied a high acceptance and feasibility of these measures if they had to be performed in the future.

In this study, we found regional variations in the perceptions toward the importance of national measures. Specifically, living in the Southern region was negatively correlated with the perceived importance of the social distancing measure group. Differences in financial management and daily habits between the South residents and other parts in Vietnam could help to explain this difference. People in Southern provinces are less likely to have available savings for risk prevention, as compared to the Northern residents (37). Social distancing or national lockdown may lead to fear of job losses, salary cuts, and unguaranteed living expenses for the family, which might be more burdensome among Southern people than those in other regions. Therefore, further national preventive measures implementation should consider the cultural background of each set to improve its feasibility and acceptability among general populations in this setting.

A recent study found that there was no difference in adherence to precautionary measures between white and blue-collar workers (4). Our findings showed that those who were white-collar workers had a lower score in the “Mandatory quarantine and personal protective equipment” compared to the health workers. Indeed, they could be classified in a lower risk of COVID-19 than health professionals, and they were allowed to work at home during the epidemic (4). Therefore, we assumed that using personal protective equipment such as facemask seems to be undervalued in this group due to their subjective sensation of safety when working from home. This reason can be applied to respondents with post-graduate education who had lower scores in both measure groups compared to those having only high school education or lower. Moreover, it means that merely educated people with higher social status underestimated mitigation strategies that touch more others than themselves. In western countries, adhering COVID 19 preventive measures was faced with skepticism by the public, even creating panic in a particular situation such as some authorities has discouraged using masks and personal protective equipment (PPE) with the reasons that its no effective protection against COVID 19 (13) and psychological barriers including using the mask and PPE as contradictory to individualism (38). Regarding cultural underpinnings aspects, using masks as well as PPE was partially restricted by the recommendation of no mask use under the context of rising political demonstration and terrorist attacks. However, this has not mentioned in literature. The former group was more likely to have stable jobs and incomes; thus, they might not be affected by the national measures. Meanwhile, people who had fixed-term employment contracts had lower levels of agreement about the importance of these measures compared to other groups. In particular, the fixed-term fulltime contract is a contractual relationship between an employee and an employer that lasts for a specified period. In Vietnam, there are two kinds of fixed-term contracts include the definite term contract of 12–36 months and the seasonal or fixed-term contract of <12 months. It may be explained that in Vietnam, people under this type of contract are at higher risk of suffering from absorbing layoffs or salary cuts without alternative income sources, or in other words, they might face substantial financial burden when they or their living areas were under quarantine. Simultaneously, those with public employment contracts could be guaranteed with full salary and monthly living expenses when occurring natural disasters or epidemics according to the Vietnamese regulations (39). Similar groups are at risk from job loss in other countries (40), which possibly reduces their positive perceptions toward the national response measures. Similarly, participants with large family size also had lower levels of agreements about the importance of “Social distancing and community screening.” These measures might cause a greater economic crisis if they lost their jobs (40).

The findings of this study indicate several implications that can be helpful in further COVID-19 and other disease epidemics in Vietnam. First, strategies to maintain and improve the perceptions of the general population toward COVID-19 and national response measures should be performed regularly to ensure that people can have sufficient preparations if the epidemic returns. Second, further preventive strategies should be contextualized to different settings to optimize their effectiveness and acceptance. Third, more studies about how people cope with the difficulties during the epidemic should be implemented in order to help to timely provide appropriate supportive interventions.

Through its rapid assessment, this study provides timely and valuable information on the perceptions and attitudes of Vietnamese people in the unique lock-down situation. However, findings of the study should be viewed in light of its limitations. First, this study used the cross-sectional design, which might hinder our ability to draw causal conclusions between people's perceptions and associated factors. Second, the snowball sampling technique used in this study possibly led to selection bias given that most of our participants were health professionals. This might limit our generalizability to the entire Vietnamese people. However, since these individuals have a central role in orienting the preventive behaviors of people in the community (41), their positive perceptions toward the national response measures is an important assurance for the success of these measures in the future. Third, our survey were administrated via online platform. Nonetheless, in the limited resource settings such as Vietnam, implementing online survey is an optimal manner to reach a large sample size quickly in various settings. Moreover, in order to increase the reliability and validity of the data, we carefully piloted the survey and presented the questionnaire in the survey platform with the most convenient way for participants to answer the questions. Fourth, given the urgent nature of the study and the challenge of participants recruitment during lockdown time, we were not able to collect a larger and more diverse sample. Thus, future studies are encouraged to consider investing more time and effort to enroll more participants with more diverse background. Further studies may also benefit from assessing the cultural aspects related to the Vietnamese's perceptions and attitudes toward COVID 19 and national response measures, in particular religious beliefs, which might be one of the factors contributing to acceptance trends of COVID-19 prevention measures in certain population groups of a multi-religious country such as Vietnam.

In conclusion, this study determined some negative perceptions and attitudes toward the national response measure to combat COVID-19 epidemic in Vietnam, including citizens who live in the Southern region, white-collar workers, post-graduate education people, and laborers with fixed-term employment contracts. Contextualized strategies to maintain and improve these perceptions should be warranted to ensure the success of the preventive measures in the future.

## Data Availability Statement

The raw data supporting the conclusions of this article will be made available by the authors, without undue reservation.

## Ethics Statement

The studies involving human participants were reviewed and approved by the study was reviewed and approved by the Review Committee of Hanoi Medical University dated 28 March 2020. The patients/participants provided their written informed consent to participate in this study.

## Author Contributions

TTPN, LN, HL, GV, MH, DN, XL, BT, TTN, QP, NT, QN, CL, RH, and CH: conceptualization and writing—review and editing. DN, TTN, QP, NT, and QN: data curation. TTPN, LN, and XL: data analysis. TTPN, LN, HL, GV, BT, and RH: methodology. HL, XL, BT, CL, RH, and CH: supervision. TTPN, LN, HL, GV, and MH: writing—original draft. GV, MH, and TTN: project administration. All authors contributed to the article and approved the submitted version.

## Conflict of Interest

The authors declare that the research was conducted in the absence of any commercial or financial relationships that could be construed as a potential conflict of interest.
